# The Book World of Medicine and Science

**Published:** 1900-05-26

**Authors:** 


### The Book World of Medicine and Science.

Supplement to the Twenty-Eighth Annual Report of the Local Government Board: Containing the Report of the Medical Officer for 1898-99. (London: Her Majesty's Stationery Office. 1899.)

We have in this report, which has just been issued, the last record of the work done under the Local Government Board by the late Sir Richard Thorne Thorne, by whom the report is signed. It is an admirable document, showing well how much good work is being done in the many branches of science which touch upon public health by the medical officers of this department. The first subject dealt with is vaccination, and we cannot but make a note of what is said in regard to so-called "insusceptibility." Of 605,807 children on whom vaccination had been performed, as many as 2,885 were certified to be insusceptible of vaccination, that is to say, they had been vaccinated unsuccessfully at least three times. But among the 3,548 primary vaccinations performed during 1898-99 by the Board's operators no such case was met with, and this number of successful primary vaccinations being added to the 107,180 similar cases referred to in the previous annual report, gives a total of 110,728 consecutive primary vaccinations performed under the Board's auspices without the occurrence of a single case of so-called insusceptibility. It is not directly said, but undoubtedly it is implied, that a great deal of very second-rate vaccination is going on, and that if vaccinators all over the country were as careful and thorough in their methods as the Board's operators, insusceptibility would be unheard of. Perhaps it would not be unfair to believe also that the same sort of care which is able to eliminate insusceptibility would, if more generally exercised, eliminate many of the "accidents," the occasional occurrence of which is made so much of by the enemies of vaccination. As usual, a considerable part of the report is devoted to the various inspections which have been made from time to time of towns and districts in which one or other of the various infectious diseases have shown an undue prevalence. In most of such cases it was enteric fever which drew the attention of the central authority to the districts in question, but the comments made upon an outbreak of measles at Burton-on-Trent are especially worth referring to. On reviewing the circumstances of this outbreak it becomes clear, we are told, that in order to successfully control measles in a borough of this description, where children suffering from the early or the incubating stages of the disease are constantly liable to come into contact with others, the measures which are most likely to avail are isolation in hospital of the children first attacked, and the strict exclusion from school of all children living in houses where the disease prevails, to be followed, if need be, by actual closure of schools. Above all things it is insisted upon that the time for preventive measures is at the very outset of the epidemic. "Not only," it is said, "can no sanitary authority be expected to maintain in readiness means of isolation for more than a limited number of cases of measles, but the value of hospital isolation for preventing the extension of this disease is generally limited to quite the initial period of an outbreak." It is pointed out that close upon 13,000 lives are annually sacrificed to measles in England and Wales, that, with the increasing aggregation of children in elementary schools, the tendency of this mortality is to increase rather than to decrease, and that the question of its control is becoming one of considerable urgency. In addition to memoranda by Dr. Bulstrode concerning the occurrence of plague on board the steamships "Caledonia" and "Golconda," a very complete report on the progress and diffusion of bubonic plague from 1879 to 1898 is given by Dr. Bruce Low. Among the tables by which this article is illustrated is one showing how definitely several epidemics of plague in Bombay alternated with the wet season. It is stated that "the subsidence of the epidemic in each of the three outbreaks of plague coincided with the advent of the rainy season," which is true in so far as this, that when the outbreak had subsided the rainy season came on; but there is nothing in the table to show any causal relation between the two events. A very important investigation by Dr. Klein on the bacillus enteritides sporogenes is reported on, although it is still incomplete. This micro-organism has already been shown to be engaged in the production of diarrhœa and cholera nostras, and the result of the experiments now detailed is to suggest that even the diarrhœa which forms so marked a symptom in some cases of typhoid fever is due to the same cause. If this should prove to be correct it would seem that typhoid with diarrhœa is to be regarded as a form of mixed infection. It must be confessed, however, that there is a great deal which is as yet very hazy in our conception of the relationship between some pathogenic organisms and the diseases which they cause, and Dr. Klein points out how little we know as to the cause of "virulence." Another very practical and important investigation made by Dr. Klein is as to the fate of pathogenic microbes in the dead body, the experiments in regard to which lend no confirmation to the general and popular belief that the microbes of infectious disease retain their vitality and power of mischief within dead and buried bodies for indefinite periods. It may be inferred from them that not only in bodies buried in coffins, but even in those buried directly (wrapped up in a piece of fabric) in earth or sand, the vitality and infectiveness of the pathogenic microbes contained in the viscera have passed away long before the outer skin has become permeable by them. Other important investigations detailed in this volume are those by Dr. Martin and Dr. Houston as to the growth of microbes, and especially of the typhoid bacillus, in soil; by Dr. Gordon on the bacteriology of scarletina; and by Dr. Copeman and Dr. Mann on the histology of vaccinia. Altogether a most interesting volume and a mine of information on matters related to public health, especially in its more scientific aspects.

